# Protective Effect of Ginsenoside Rb1 against Intestinal Ischemia-Reperfusion Induced Acute Renal Injury in Mice

**DOI:** 10.1371/journal.pone.0080859

**Published:** 2013-12-04

**Authors:** Qian Sun, Qing-tao Meng, Ying Jiang, Hui-min Liu, Shao-qing Lei, Wa-ting Su, Wei-na Duan, Yang Wu, Zheng-yuan Xia, Zhong-yuan Xia

**Affiliations:** 1 Department of Anesthesiology, Renmin Hospital of Wuhan University, Wuhan, Hubei, China; 2 Department of Anesthesiology, The University of Hong Kong, Hong Kong, China; 3 Department of Anesthesiology, Affiliated Hospital of Guangdong Medical College, Zhanjiang, Guangdong, China; University College London, United Kingdom

## Abstract

Ginsenoside Rb1 (RB1), the most clinically effective constituent of ginseng, possesses a variety of biological activities. The objectives of this study were to investigate the protective effects of RB1 and its underlying mechanism on renal injury induced by intestinal ischemia-reperfusion (IIR) in mice. RB1 was administered prior to inducing IIR achieved by occluding the superior mesenteric artery for 45 min followed by 120 min of reperfusion. All-trans-retinoic acid (ATRA) was used as an inhibitor of NF-E2-related factor-2 (Nrf2) signaling. Adult male C57BL/6J mice were randomly divided into six groups: (1) sham group, (2) IIR group, (3) RB1 group, (4) sham + ATRA group, (5) IIR + ATRA group, and (6) RB1 + ATRA group. Intestinal histology and pathological injury score were observed. Intestinal mucosal injury was also evaluated by measuring serum diamine oxidase (DAO). Renal injury induced by IIR was characterized by increased levels of histological severity score, blood urea nitrogen (BUN), serum creatinine (Scr) and neutrophil gelatinase-associated lipocalin (NGAL), which was accompanied with elevated renal TUNEL-positive cells and the Bcl-2/Bax expression ratio. RB1 significantly reduced renal injury and apoptosis as compared with IIR group, which was reversed by ATRA treatment. Immunohistochemistry and Western blot analysis demonstrated that RB1 significantly upregulated the protein expression of heme oxygenase-1 (HO-1) and Nrf2, which were attenuated by ATRA treatment. Taken together, these results suggest that the protective effects of RB1 pretreatment against renal injury induced by IIR are associated with activation of the Nrf2/ anti-oxidant response element (ARE) pathway.

## Introduction

Intestinal ischemia-reperfusion (IIR) induces a range of adverse responses, ranging from relatively subtle changes in mucosal capillary permeability to gross trans-mural infarction, depending on both the severity and duration of the insult. [1] Translocation of bacteria and toxins through a leaky gut mucosa may amplify or perpetuate systemic inflammation and oxidative stress, leading to multiple organ failure and death in critically ill patients. [2] Recent studies have shown that IIR causes significant oxidative injury in rat renal parenchyma, consisting of severe alterations observed at the level of subcellular renal structures, and is associated with significant failure of kidney function. [3], [4]

Ginsenoside Rb1 (RB1), the most clinically effective constituent of ginseng, possesses a variety of biological activities including anti-oxidant, anti-inflammatory and anti-apoptosis effects. [5], [6] RB1 pre-conditioning has been shown to limit renal ischemia-reperfusion injury and interstitial fibrosis formation and attenuate renal apoptosis and oxidative damage. [7], [8] Attenuation of apoptosis and oxidative stress are known to play important roles in the renal protective effects mediated by a variety of treatment interventions. Recent data indicate that the NF-E2-related factor-2/anti-oxidant response element (Nrf2/ARE) regulatory pathway plays a central role in the protective effect against oxidative and apoptotic damage. [9], [10] Hence, it was of interest to determine whether RB1 can protect against renal injury resulted from IIR through the Nrf2/ARE pathway.

In the present study, we examined the protective effects of RB1 against IIR-induced renal injury and explored the underlying mechanisms. Renal damage was assessed by histology, measurement of biomarkers that reflect renal damage, and quantitation of apoptosis and the oxidative stress response. The results showed that there was significant protection from IIR- induced renal injury by RB1, which was reversed by all-trans-retinoic acid (ATRA), an established inhibitor of the Nrf2/ARE pathway, and that the protection involved changes in oxidative stress response pathways.

## Materials and Methods

### Materials

Adult male C57BL/6J mice, weighing 25 ± 3 g, were obtained from Hunan Slac Jd Laboratory Animal Co. Ltd. (Hunan, China). The experimental protocol used in this study was reviewed and approved by the Animal Care and Use Committee of Wuhan University. This was in accordance with the Guide for the Care and Use of Laboratory Animals by the National Institutes of Health (NIH Publication No. 80–23). RB1 (purity by high-performance liquid chromatography >98%) was purchased from the National Institute for the Control of Pharmaceutical and Biological Products (Beijing, China) and dissolved in saline. Antibodies for Nrf2, heme oxygenase-1 (HO-1), Bcl-2 and Bax were purchased from Santa Cruz Biotechnology, Inc. (Santa Cruz, CA, USA). ATRA was purchased from Sigma-Aldrich Trading Co. Ltd. (Shanghai, China). All other chemicals were obtained from commercial sources and were of the highest grade available.

### Experimental protocol

Animals were anesthetized using intra-peritoneal injections of sodium pentobarbital (50 mg/kg). The IIR model was established by occlusion of the superior mesenteric artery (SMA) as described. [11] Mice were randomly assigned into one of six experimental groups (n = 8 per group) as follows: (1) a control group (Sham group) that underwent isolation of the SMA without occlusion; (2) IIR group being subjected to 45 min of intestinal ischemia and 2 h of reperfusion after the SMA had been isolated, and received 10 ml/kg saline 10 min before reperfusion (IIR group); (3) RB1 treated group (6 mg/ml, dissolved in saline, 10 ml/kg i.p., 10 min before reperfusion) (RB1 group); (4) sham-operated mice treated with ATRA (ATRA + Sham group); (5) IIR mice treated with ATRA (ATRA + IIR group); and (6) sham-operated mice treated with both ATRA and RB1 (ATRA + RB1 group). Additionally, the mice in groups 4, 5 and 6 were fed a vitamin A-deficient diet (Special Diet Service) for six weeks before the experiment. During the last two weeks, they received ATRA (2 mg/ml, dissolved in saline, 10 ml/kg i.p. daily for two weeks before the operation). [12] Kidney, intestine and blood samples were collected at the end of the reperfusion period.

### Histopathological assessment of intestines

After reperfusion, 1 cm of small intestine without adipose tissue was taken from the same place at the distal end of ileum, and fixed in 4% formaldehyde. After embedding in paraffin, 4-µm sections were stained with hematoxylin and eosin before assessment by light microscopy (original magnification ×200, Olympus BX50; Olympus Optical, Tokyo, Japan).

Using the improved Chiu score [13] method to evaluate intestinal mucosal damage, higher scores are interpreted to indicate more severe damage. Criteria of Chiu grading system consists of 5 subdivisions according to the changes of villus and gland of intestinal mucosa: grade 0, normal mucosa; grade 1, development of subepithelial Gruenhagen's space at the tip of villus; grade 2, extension of the space with moderate epithelial lifting; grade 3, massive epithelial lifting with a few denuded villi; grade 4, denuded villi with exposed capillaries; and grade 5, disintegration of the lamina propria, ulceration and hemorrhage.

### Detection of diamine oxidase activity in serum

Blood samples were collected at the end of reperfusion and centrifuged at 3,000 g, for 10 min at 4°C. Serum was separated and stored at −20°C. Serum diamine oxidase (DAO) was detected using a chemical assay kit (Nanjing Jiancheng Biochemicals Ltd, Nanjing, China) according to the manufacturer's protocol.

### Histopathological assessment of kidneys

The left kidney was removed, cut into sections, and fixed in 4% formaldehyde. After embedding in paraffin, 4-µm sections were stained with hematoxylin and eosin before assessment by light microscopy (original magnification ×200, Olympus BX50; Olympus Optical, Tokyo, Japan).

Histologic assessment of tubular necrosis was determined semi-quantitatively using a method modified from McWhinnie et al. [14] Scores were: 0 = normal histology; 1 = tubular cell swelling, brush border loss, nuclear condensation, with up to one-third of the tubular profile showing nuclear loss; 2 = same as for score 1, but greater than one-third and less than two-thirds of the tubular profile showing nuclear loss; and 3 = greater than two-thirds of the tubular profile showing nuclear loss.

### Measurement of blood urea nitrogen (BUN) and serum creatinine (Scr)

Blood samples were collected at the end of reperfusion and centrifuged at 3,000 g for 10 min at 4°C. Serum was separated and stored at −20°C. BUN and Scr levels were measured using an Olympus automatic analyzer (AU5400; Olympus Optical, Tokyo, Japan).

### Measurement of serum neutrophil gelatinase-associated lipocalin (NGAL)

Blood samples were collected at the end of reperfusion and centrifuged at 3,000 g, for 10 min at 4°C. Serum was separated and stored at −20°C. NGAL levels were measured using a NGAL assay kit (Boster Biological Technology, Wuhan, China) according to the manufacturer's instructions.

### Determination of renal apoptosis

A terminal deoxynucleotidyltransferase dUTP nick-end labeling (TUNEL) assay was used to assess renal apoptosis with an apoptosis detection kit (Boster Biological Technology) according to the manufacturer's instructions. For each slide, ten fields were randomly chosen, with TUNEL-positive cells displaying brown staining within the nucleus of apoptotic cells. Apoptotic cells were quantified under high-power magnification by an investigator in a blinded manner and the apoptotic index was calculated (the number of TUNEL-positive or apoptotic cells/total number of cells counted×100).

### Renal HO-1 and Nrf2 immunohistochemical assays

Paraffin-embedded renal sections were stained using the streptavidin-biotin complex (SP×200) immunohistochemistry technique for HO-1 and Nrf2 detection. Brown staining in the cytoplasm and/or nucleus was considered as an indicator of positive expression. Results were evaluated semi-quantitatively with Image-Pro® plus version 6.0 software according to optical density values correlating with positive expression.

### Western blot analysis

Cytoplasmic and nuclear proteins were extracted from frozen renal tissues with a nuclear extraction kit according to the manufacturer's instructions. An equal amount of protein was loaded on to 12% SDS-PAGE at 100 V for 3 h. After electrophoresis, proteins were transferred onto PVDF membranes at 200 mA for 2 h. The transferred membranes were incubated overnight at 4°C with rabbit anti-mouse polyclonal antibodies for HO-1, Nrf2, Bcl-2 or Bax (each at 1:800 dilutions) in Tris phosphate-buffered saline (TBS-T) containing 5% skimmed milk. After washing three times in TBS-T, membranes were incubated with anti-rabbit IgG conjugated to horseradish peroxidase at a dilution of 1:2,000 in TBS-T containing 5% skimmed milk for 2 h at room temperature. The immunoreactive bands were visualized with enhanced chemiluminescence and captured on X-ray film. Optical density of the bands was measured using the BandScan imaging analysis system.

### Statistical analysis

Mean ± SEM values were calculated to summarize all outcome measurements. One-way analysis of variance and the Duncan's multiple range method were used to compare significant differences among the groups. The level of significance was set at *P*<0.05 for all statistical tests.

## Results

### Histopathological assessment of intestines

**Figure 1:**
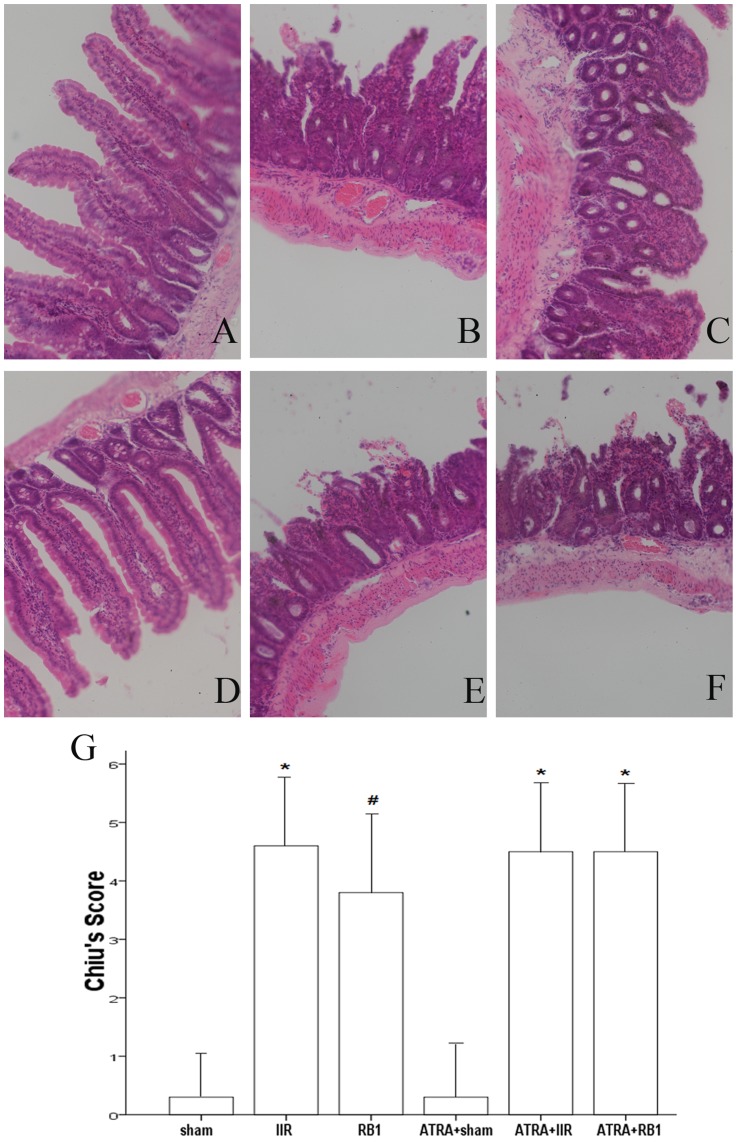
Intestinal histologic evaluation in the various treatment groups. *A–F*, Histopathologic changes of the small-intestinal mucosa were observed under light microscopy (original magnification, ×200). In the Sham and ATRA+Sham groups (*A*, *D*), normal intestinal mucosa was seen. In contrast, severe mucosal damage was observed in the IIR, ATRA+IIR and ATRA+RB1 groups (*B*, *E* and *F*). Compared with the IIR group, only mild damage in intestinal architecture was seen in the RB1 group (*C*). *G*, Changes in intestinal mucosal Chiu's scores. A minimum of five randomly chosen fields from each mouse were evaluated and averaged to determine mucosal damage. Data are expressed as means ± SEM (n = 6, *P<0.05 vs. Sham group, #*P*<0.05 vs. IIR group).

In Fig. 1*A-F*, normal villi were observed in the Sham and ATRA + Sham groups. By contrast, the IIR and ATRA + IIR groups demonstrated edema in the villi and apparent inflammatory cells infiltration, and many intestinal villi were severed and denuded. In addition, the gap between epithelial cells significantly increased and capillaries and lymph vessels were markedly dilated. Significant amelioration of histological injury was observed in the RB1-treated groups, while the ATRA + RB1 group exhibited the same extent of injury as seen in the IIR group.

In parallel with the mucosal morphologic changes, Chiu's score in the IIR group was higher than that in the Sham group (*P*<0.01). This increase was significantly reduced by administration of RB1 (*P*<0.01). However, there was no statistically significant difference in Chiu's score between the IIR group and the ATRA + RB1 group (*P*>0.05) (Fig. 1*G*).

### Evaluation of intestinal mucosal injury

DAO (U/L serum) is an enzyme synthesized primarily by gastrointestinal mucosal cells, and the serum level of DAO has been used as an indicator of the integrity and functional mass of the intestinal mucosa. The serum DAO activity was increased in groups IIR (22.61 ± 1.59), ATRA+IIR (21.44 ± 1.34) and ATRA+RB1 (23.21 ± 1.06) and was decreased in groups treated with RB1 (16.60 ± 1.24) (all P<0.05).

### Histopathological assessment of kidneys

**Figure 2:**
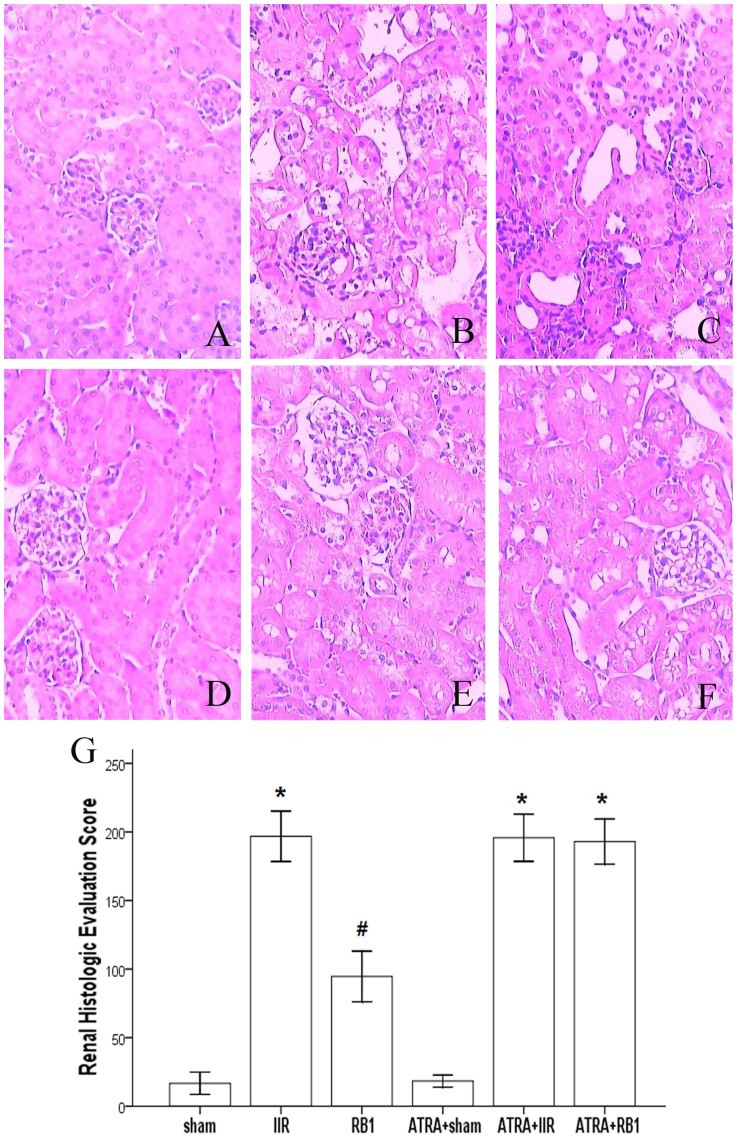
Renal histologic evaluation in the various treatment groups. *A–F*, Histopathologic changes of the kidney were observed under light microscopy (original magnification, ×200). In the Sham and ATRA+Sham groups (*A*, *D*), normal glomerular and kidney tubules were seen. In contrast, severe damage was observed in the IIR, ATRA+IIR and ATRA+RB1 groups (*B*, *E* and *F*). Compared with the IIR group, only mild damage in renal architecture was seen in the RB1 group (*C*). *G*, Changes in total severity score measured from the kidney. A minimum of five randomly chosen fields from each mouse were evaluated and averaged to determine mucosal damage. Data are expressed as means ± SEM (n = 6, *P<0.05 vs. Sham group, #*P*<0.05 vs. IIR group).

RB1 attenuated renal histological injury at 2 h after intestinal reperfusion. Renal tubules in the IIR group showed pathological changes, including edema, necrosis and vacuolization (Fig. 2*A–F*). Significant amelioration of histological edema, necrosis and vacuolization was observed in the RB1-treated groups, while the ATRA + RB1 group exhibited the same extent of injury as the IIR group.

When compared with the total severity score measured from kidneys obtained from Sham animals, IIR resulted in a significant increase in total severity score (*P*<0.01). This increase was significantly reduced by administration of RB1 (*P*<0.01). However, there was no statistically significant difference in total severity score when the IIR group was compared with the ATRA + RB1 group (*P*>0.05) (Fig. 2*G*).

### Assessment of BUN and Scr levels

**Figure 3:**
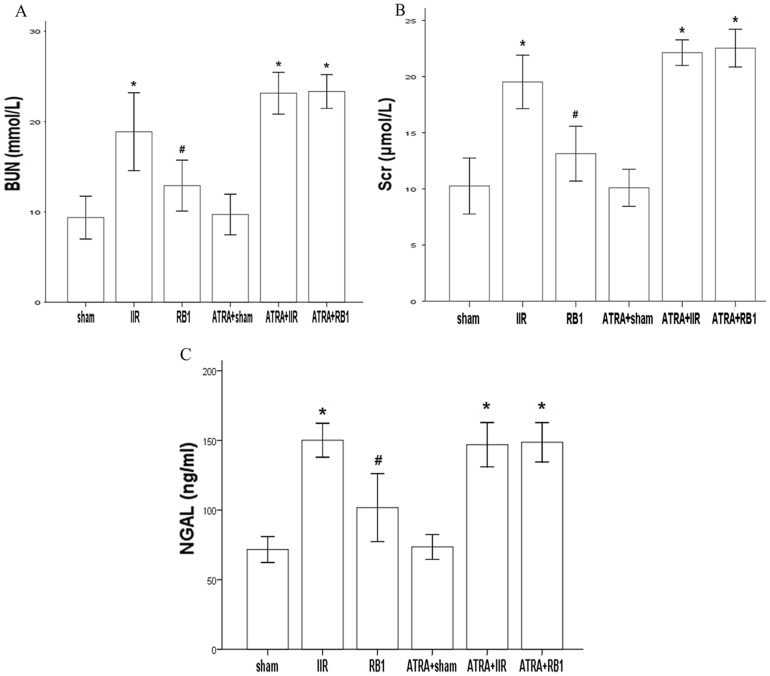
BUN, Scr and NGAL levels in the different treatment groups. *A*, Changs of BUN level. *B*, Changs of Scr level. *C*, Changs of NGAL level. Data are expressed as means ± SEM (n = 8, ^*^ *P*<0.05 vs. Sham group, ^#^ *P*<0.05 vs. IIR group).

BUN (mmol/L) and Scr (µmol/L) levels were measured to evaluate the extent of renal injury associated with IIR. IIR significantly increased BUN and Scr levels compared with the Sham group (*P*<0.01). Treatment with RB1 markedly decreased BUN and Scr levels compared with the IIR group (*P*<0.01). However, there was no difference in BUN and Scr levels compared with the ATRA+RB1 group (*P*>0.05) (Fig. 3*A, B*).

### Assessment of serum NGAL levels

Serum NGAL levels (ng/ml) were measured to further evaluate the effects of RB1 on renal injury associated with IIR. Following a 45 min period of intestinal ischemia, reperfusion significantly increased serum NGAL levels in the IIR group compared with the Sham group (*P*<0.01). Treatment with RB1 markedly decreased serum NGAL levels compared with the IIR group (*P*<0.01). However, there was no difference in NGAL levels compared with the ATRA+RB1 group (*P*>0.05) (Fig. 3*C*).

### Effect of RB1 on renal apoptosis

**Figure 4:**
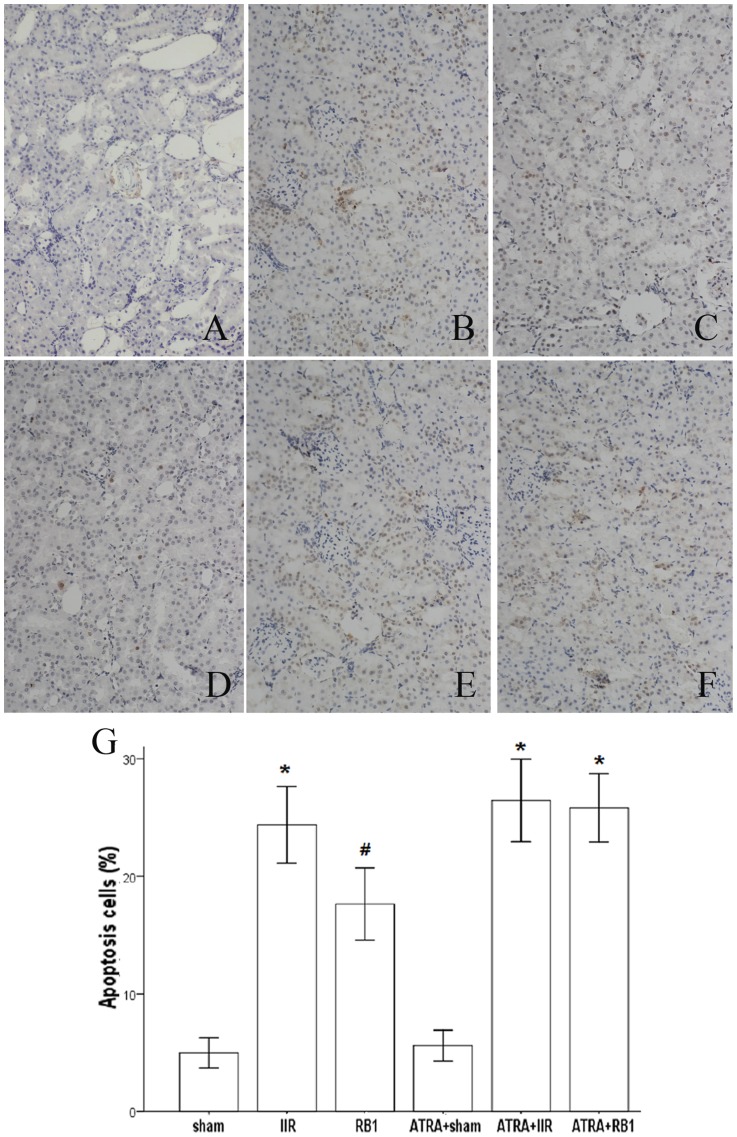
Changes of the renal tubular epithelial cell apoptosis as measured by TUNEL staining. *A–F*, Representative photomicrographs of TUNEL staining from the different treatment groups (original magnification, ×200). In the Sham and ATRA+Sham groups (*A*, *D*), little brown staining was seen. In contrast, many TUNEL-positive cells displaying brown staining within the nucleus of apoptotic cells were observed in the IIR, ATRA+IIR and ATRA+RB1 groups (*B*, *E* and *F*). Compared with the IIR group, the number of TUNEL-positive cells displaying brown staining was reduced in the RB1 group (*C*). *G*, Apoptotic cells were quantified under high-power magnification by an investigator in a blinded manner and the apoptotic index ( = the number of TUNEL-positive or apoptotic cells/total number of cells counted×100) was calculated. Results are expressed as means ± SEM of apoptotic cells (%) (n = 4, *P<0.05 vs. Sham group, #*P*<0.05 vs. IIR group).

TUNEL staining was used to evaluate the anti-apoptotic effect of RB1. IIR significantly increased the number of TUNEL-positive cells in mouse kidneys as compared to the Sham (*P*<0.05). RB1 treatment reduced the number of TUNEL-positive cells in the kidneys of mice subjected to IIR (*P*<0.05). Pretreatment with ATRA eliminated the effects of RB1 in reducing the number of TUNEL-positive cells (*P*<0.05 for IIR vs. ATRA + RB1 group) (Fig. 4).

### Effects of RB1 on Bcl-2/Bax expression ratio in renal tissues

**Figure 5:**
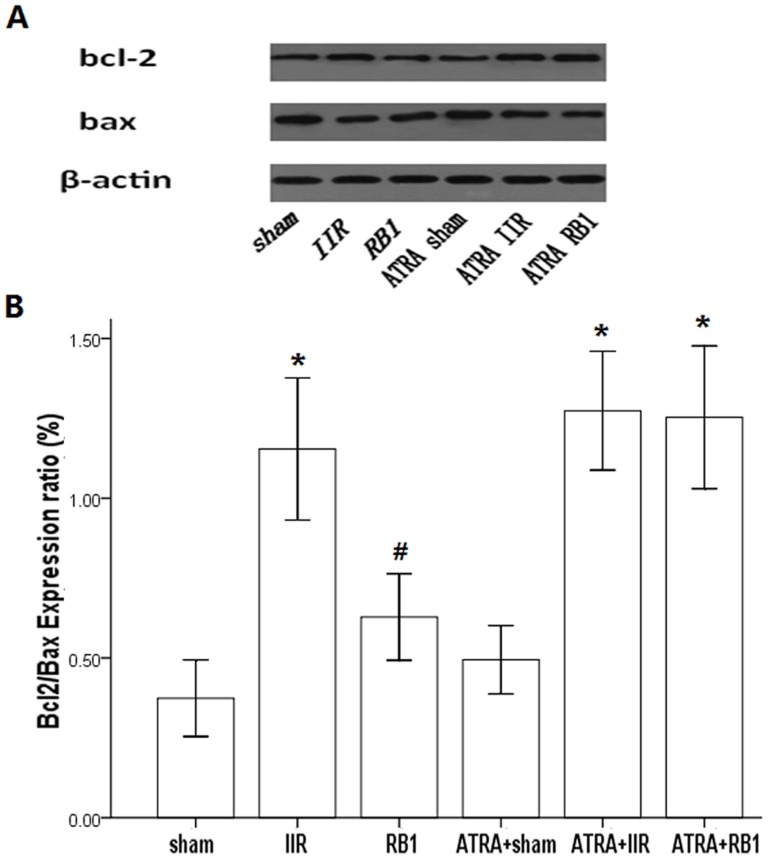
The expression of Bcl-2 and Bax in renal tissues in the different treatment groups by Western blot analysis. *A*, Western blot analysis for the presence of Bcl-2 and Bax in renal tissues. β-actin was used as the protein loading control. *B*, Bcl-2 and Bax protein contents were detected by densitometry. Data are expressed as means ± SEM of Bcl-2/Bax expression ratio (n = 6, *P<0.05 vs. Sham group, #*P*<0.05 vs. IIR group).

Western blot analysis revealed that the expression ratio of Bcl-2/Bax in the IIR group was higher compared with that in the Sham group (*P*<0.05). RB1 attenuated the increase in the expression ratio of Bcl-2/Bax compared with that in the IIR group (*P*<0.05). Pre-treatment with ATRA eliminated the effects of RB1 in reducing the expression ratio of Bcl-2/Bax (*P*<0.05, IIR vs. IIR + RB1 group) (Fig. 5).

### Effects of RB1 on HO-1 and Nrf2 expression in renal tissues assessed by immunohistochemical assay

**Figure 6:**
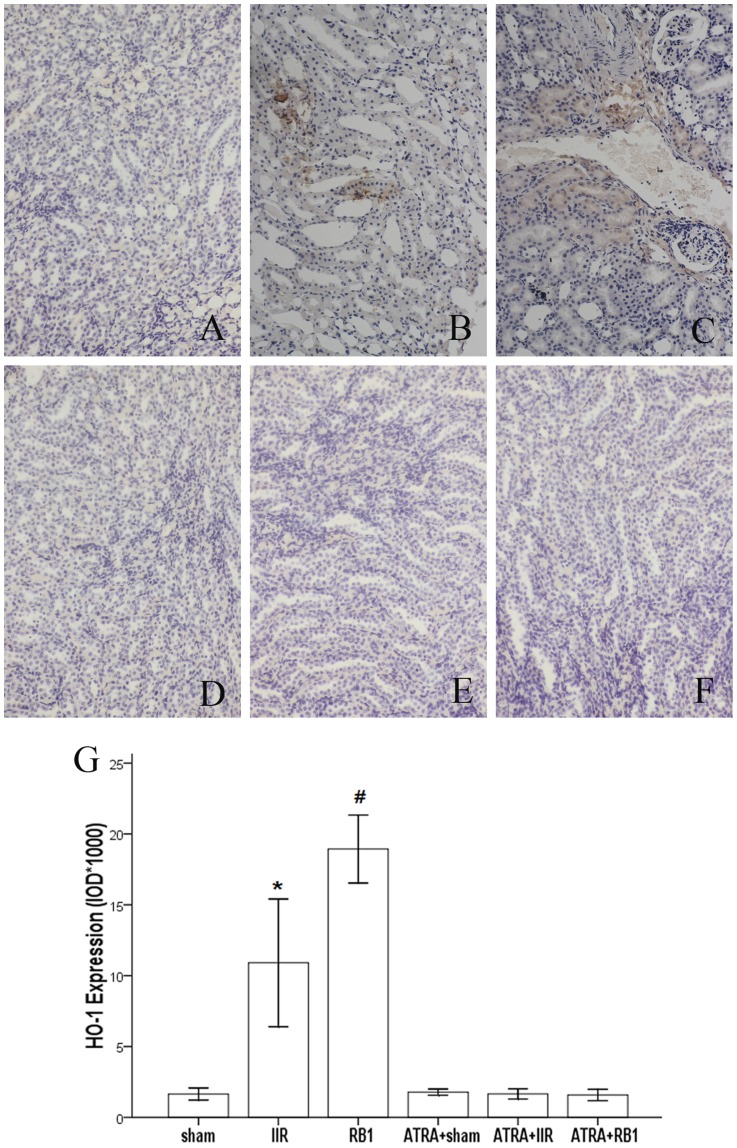
Expression of HO-1 in renal tissues assessed by immunohistochemistry. *A–F*, Representative photomicrographs of streptavidin-perosidase staining from the different treatment groups (original magnification, ×200). In the Sham and ATRA+Sham groups (*A*, *D*), little brown staining was seen. In contrast, many positive cells displaying brown staining within the cytoplasm of renal cells were observed in the IIR, ATRA+IIR and ATRA+RB1 groups (*B*, *E* and *F*). Compared with the IIR group, the number of positive cells displaying brown staining was reduced in the RB1 group (*C*). *G*, Positive expression was evaluated semi-quantitatively by optical density (IOD×10^3^). Data are expressed as means ± SEM (n = 8, *P<0.05 vs. Sham group, #*P*<0.05 vs. IIR group).

Analysis of the expression of HO-1 in the Sham group showed sparse brown immunostaining in the cytoplasm while there was significant, positive expression of HO-1, as indicated by dense brown staining in the cytoplasm in the IIR group (*P*<0.01). Compared with the IIR group, the positive staining of HO-1 expression increased significantly in the cytoplasm in the RB1 group (*P*<0.01). However, there was no alteration in HO-1 staining in the ATRA+RB1 group, compared to the IIR group (*P*>0.05) (Fig. 6).

**Figure 7:**
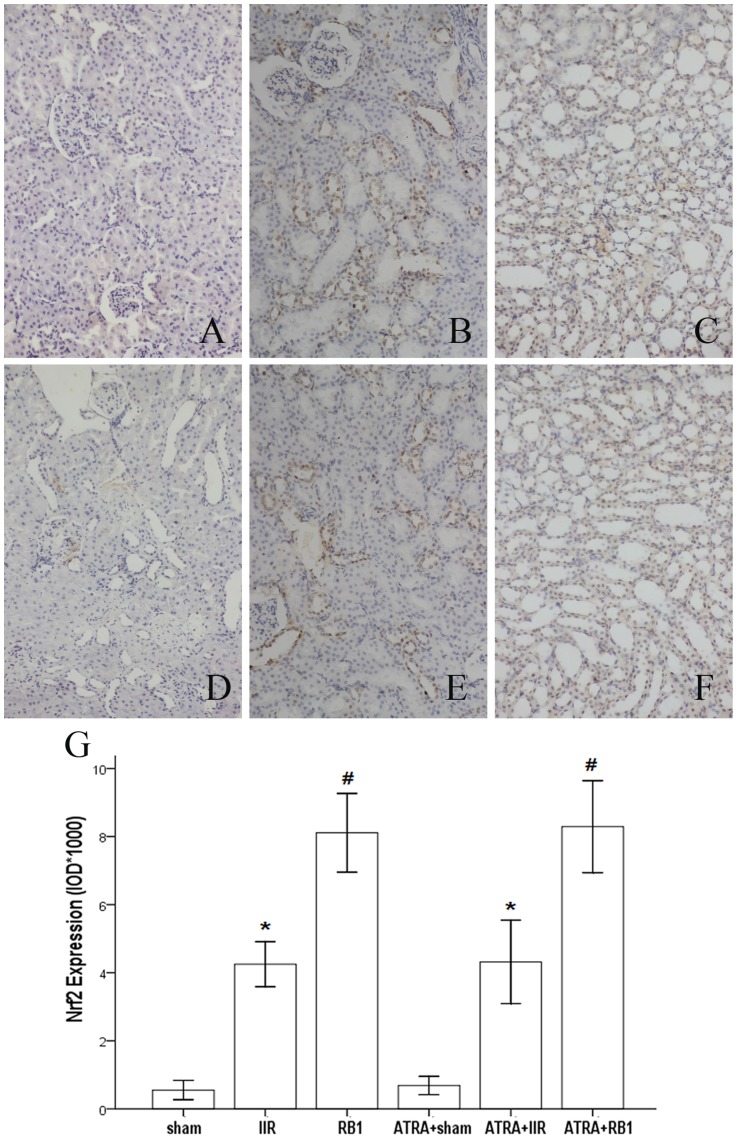
Expression of Nrf2 in renal tissues assessed by immunohistochemistry. *A–F*, Representative photomicrographs of streptavidin-perosidase staining from the different treatment groups (original magnification, ×200). In the Sham and ATRA+Sham groups (*A*, *D*), little brown staining was seen. In contrast, many positive cells displaying brown staining within the cytoplasm and nuclei of renal cells were observed in the IIR, ATRA+IIR and ATRA+RB1 groups (*B*, *E* and *F*). Compared with the IIR group, the number of positive cells displaying brown staining was reduced in the RB1 group (*C*). *G*, Positive expression was evaluated semi-quantitatively by optical density (IOD×10^3^). Data are expressed as means ± SEM (n = 8, *P<0.05 vs. Sham group, #*P*<0.05 vs. IIR group).

The expression of Nrf2 in the Sham group showed light brown immunostaining in the cytoplasm and no staining in the nuclei. However, in the IIR group, there was significantly augmented expression of Nrf2, as indicated by dense brown staining in the cytoplasm and nuclei (*P*<0.01). Compared with the IIR group, the positive staining for Nrf2 expression increased significantly in the RB1 group (*P*<0.01). However, the ATRA-treated groups exhibited the same alteration in staining as the normal groups (Fig. 7).

### Effects of RB1 on Nrf2 and HO-1 expression in renal tissues

**Figure 8:**
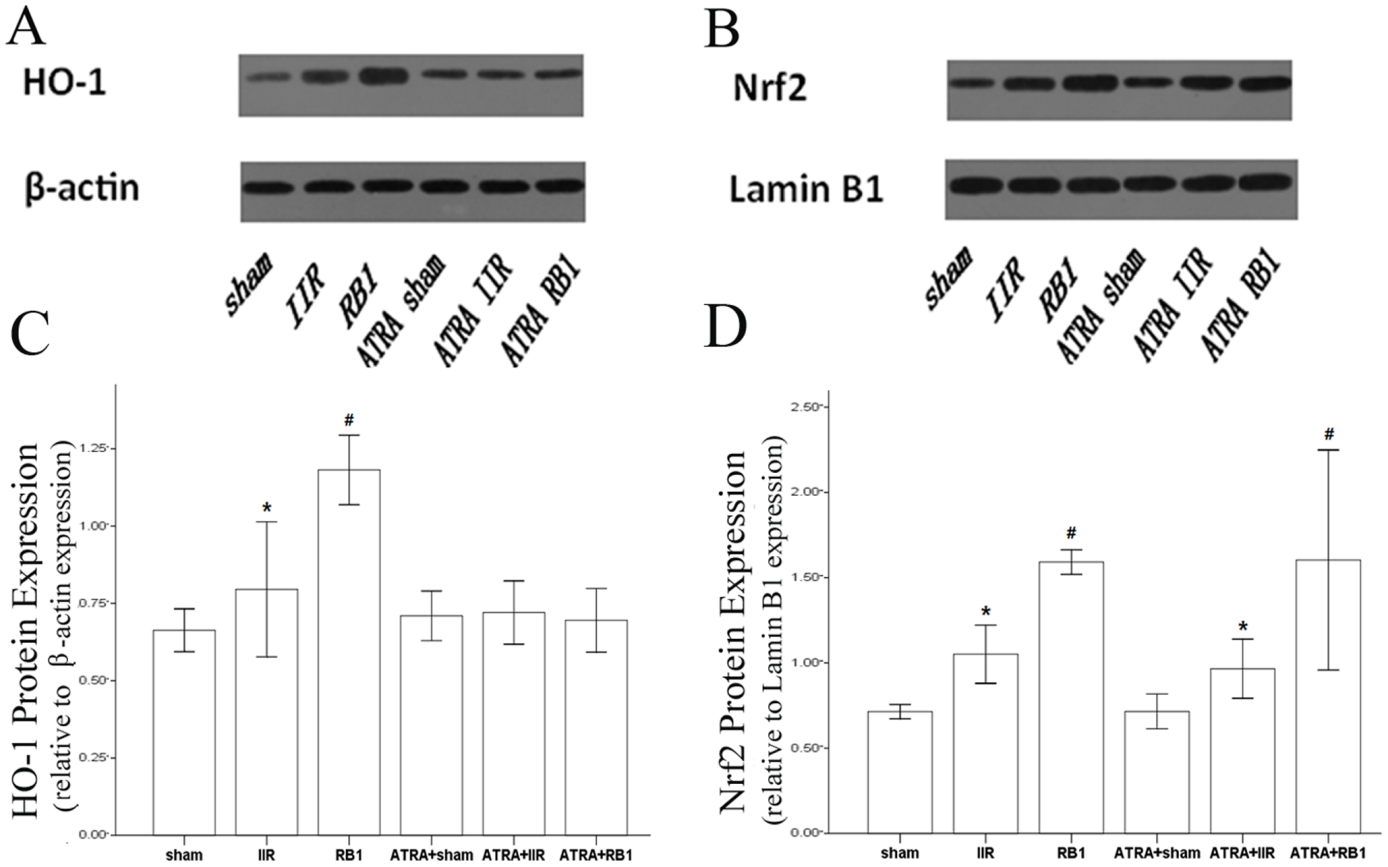
Expression of HO-1 and Nrf2 in renal tissues as assessed by Western blot. *A & B*, Western blot analysis for the presence of HO-1 in cytoplasmic proteins and Nrf2 in nuclear proteins. β-actin and Lamin B1 were used as the protein loading controls for HO-1 and Nrf2, respectively. *C & D*, HO-1 and Nrf2 protein contents were detected by densitometry. Data are expressed as means ± SEM (n = 6, *P<0.05 vs. Sham group, #*P*<0.05 vs. IIR group).

Western blot analysis showed weak signals for Nrf2 and HO-1 in the kidneys of the Sham group. In contrast, significant increases in protein expression for Nrf2 and HO-1 were found in the IIR group (*P*<0.01). Compared with the IIR group, the Western blot signals were intensified in renal tissues from the RB1-treated group (*P*<0.01). However, no difference in HO-1 expression was observed among the ATRA-treated groups (*P*>0.05) while Nrf2 expression in renal tissue from the ATRA-treated groups exhibited the same changes in Western blot intensity as that of the Sham group (Fig. 8).

## Discussion

The main findings of this study are that the Nrf2/ARE pathway was activated in IIR-treated mouse kidney, pre-treatment with RB1 enhanced Nrf2 translocation to the nucleus in renal tissues of mice subjected to IIR, and RB1 treatment reduced renal injury and apoptosis. Furthermore, treatment with ATRA, which is an established inhibitor of the Nrf2/ARE pathway, reversed the renal-protective effects of RB1, as indicated by decreased activation of the Nrf2/ARE pathway. These findings suggest that RB1 may confer its protective effect by activating the Nrf2/ARE pathway.

Recent studies have shown that IIR significantly aggravates renal injury. [3], [4], [15] As pre-conditioning plays a role through a number of signaling pathways, the renal-protective effects in IIR-treated mice might be enhanced by activation of the Nrf2/ARE pathway. [16] In the present study, Nrf2 expression was significantly higher in the IIR group than that observed in the Sham group, which suggests the activation of the Nrf2/ARE pathway in the kidneys following IIR treatment. Of particular note is the observation that RB1 enhanced post-ischemic renal expression levels of Nrf2, which we speculate is the major mechanism whereby RB1 reduced renal injury and apoptotic cell death given that the protective effect of RB1 was abolished by ATRA with concomitant reduction of Nrf2 expression.

It is well known that damage to the intestinal mucousal membrane subsequent to IIR can cause systemic inflammation and oxidative stress. [2] RB1 has been shown to improve hepatic function and raise the survival rate in rats suffering from IIR. [17] Previous studies have not investigated whether or not RB1 can reverse changes in renal function caused by IIR. In this study, RB1 treatment resulted in a decrease of BUN, Scr and NGAL levels at the end of reperfusion, which indicated improved renal function. This protection was further corroborated by assessment of effects on renal histopathology. The results in the present study clearly showed that RB1 treatment exerts renal protective effects in IIR-treated mice.

Activation of Nrf2/ARE has been shown to play an important protective role in both renal ischemic injury and in IIR. [18]–[21] To gain insight into the potential mechanisms responsible for the protective effect afforded by RB1 against IIR-induced apoptosis, we further evaluated the role of the Nrf2/ARE pathway. Our results showed that there were significant increases in expression of Nrf2 and HO-1 after reperfusion in the kidneys of RB1-treated mice. More importantly, pretreatment with ATRA, an Nrf2/ARE inhibitor, antagonized Nrf2 through an interaction with the retinoic acid receptor alpha, which apparently prevented Nrf2 from binding to the ARE. [12] ATRA abolished the renal-protective effect of RB1, as demonstrated by reversal of the RB1-induced decrease in apoptosis and renal histopathological injury. Furthermore, whereas IIR was associated with a significant increase in the serum NGAL, a biochemical marker of acute renal injury, [22]–[24] RB1 prevented this increase and treatment with ATRA reversed the effect of RB1 on serum NGAL. These results corroborate the conclusion that the Nrf2/ARE pathway contributes to the RB1-elicited renal-protective effect against IIR injury in mice.

There are several reports assessing the protective effects of ginseng on ischemia reperfusion injury. [25]–[28] Our previous studies showed that RB1 could attenuate oxidative stress, which is thought to play the key role in protecting various organs from ischemia-reperfusion injury. [27], [28] Recently, Hwang et al. [29] demonstrated that RB1 augments cellular anti-oxidant defenses through endoplasmic reticulum-dependent HO-1 induction via the Gbeta1/PI3K/Akt-Nrf2 pathway, thereby protecting cells from oxidative stress. Indeed, induction of HO-1 expression via the PI3K/Akt-Nrf2 has recently been shown to play key roles in antioxidant mediated protection against organ ischemia-reperfusion injury. [30], [31] Therefore, we postulate that activation of the Nrf2 pathway with the subsequent enhancement of HO-1 expression play an important role in attenuating IIR-induced remote organ kidney injury in mice, although this hypothesis needs further validation in both *in vitro* and *in vivo* studies.

Ischemia-reperfusion enhances Nrf2 dissociation from Keap1, translocation to the nucleus, binding to the ARE, and activation of phase 2 detoxifying and antioxidant genes. [32], [33] The Nrf2/ARE pathway affects cell survival through a variety of substrates, including apoptotic proteins such as Bcl-2 and Bax [34], [35] and phase 2 enzymes such as HO-1. [36], [37] HO-1, which is considered a stress protein, is regarded as a sensitive and reliable indicator of cellular oxidative stress. [38] The present study confirmed the adverse effects of IIR on the Bcl-2/Bax ratio and HO-1 expression, the prevention of these effects by RB1, and elimination of that preventive effect of RB1 by ATRA. Hence, activation of the Nrf2/ARE pathway with the subsequent enhancement of HO-1 expression and reduction of IIR-induced renal apoptotic cell death may represent the major or key mechanism whereby RB1 confers its protection against IIR -induced renal injury.

In conclusion, our present study indicates that treatment of mice with RB1 after IIR reduces renal apoptosis and alleviates renal dysfunction at least in part through the Nrf2/ARE signaling pathway. RB1 may provide a novel therapeutic strategy for treatment of IIR-induced remote organ injury.

## References

[1] Haglund U, Bulkley GB, Granger DN (1987) On the pathophysiology of intestinal ischemic injury. Clinical review. Acta Chir Scand 153: 321–4. 3310486

[2] Schwarz B, Salak N, Hofstötter H, Pajik W, Knotzer H, et al (1999) Intestinal ischemic reperfusion syndrome: pathophysiology, clinical significance, therapy. Wien Klin Wochenschr 111: 539–48. 10467640

[3] Yurdakan G, Tekin IO, Comert M, Acikgoz S, Sipahi EY (2012) The presence of oxidized low-density lipoprotein and inducible nitric oxide synthase expression in renal damage after intestinal ischemia reperfusion. Kaohsiung J Med Sci 28: 16–22. 22226057 10.1016/j.kjms.2011.06.030 PMC11916904

[4] Kazantzidou D, Tsalis K, Vasiliadis K, Kaldrymidou H, Papageorgiou G, et al (2010) Alanine-glutamine dipeptide pretreatment protects rat renal function from small intestine ischemia-reperfusion injury. Minerva Chir 65: 515–25. 21081863

[5] Jia JM, Wang ZQ, Wu LJ, Wu YL (2008) Advance of pharmacological study on ginsenoside Rb1. Zhongguo Zhong Yao Za Zhi 33: 1371–7. 18837333

[6] Cheng Y, Shen LH, Zhang JT (2005) Anti-amnestic and anti-aging effects of ginsenoside Rg1 and Rb1 and its mechanism of action. Acta Pharmacologica Sinica 26: 143–9. 15663889 10.1111/j.1745-7254.2005.00034.x

[7] Zhu MX, Ran B, Feng ZQ, Pan QW (2009) Effects of Rb1 and Rg1 on the expression of Bcl-2/bax, Bax in apoptosis of HK-2 cells induced by the serum of kidney ischemia/reperfusion. Zhongguo Ying Yong Sheng Li Xue Za Zhi 25: 496–9. 21158042

[8] Xie XS, Liu HC, Yang M, Zuo C, Deng Y, et al (2009) Ginsenoside Rb1, a panoxadiol saponin against oxidative damage and renal interstitial fibrosis in rats with unilateral ureteral obstruction. Chin J Integr Med 15: 133–40. 19407952 10.1007/s11655-009-0133-9

[9] Taguchi K, Motohashi H, Yamamoto M (2011) Molecular mechanisms of the Keap1–Nrf2 pathway in stress response and cancer evolution. Genes Cells 16: 123–40. 21251164 10.1111/j.1365-2443.2010.01473.x

[10] Tkachev VO, Menshchikova EB, Zenkov NK (2011) Mechanism of the Nrf2/Keap1/ARE signaling system. Biochemistry (Mosc) 76: 407–22. 21585316 10.1134/s0006297911040031

[11] Mutlu G, Abbasoğlu L, Doğru-Abbasoğlu S, Solakoğlu S, Bulut M (2002) Morphologic changes and lipid peroxidation in renal tissuess of young rats following intestinal ischemia-reperfusion. Pediatr Surg Int 18: 337–40. 12415350 10.1007/s00383-002-0834-z

[12] Wang XJ, Hayes JD, Henderson CJ, Wolf CR (2007) Identification of retinoic acid as an inhibitor of transcription factor Nrf2 through activation of retinoic acid receptor alpha. Proc Natl Acad Sci U S A 104: 19589–94. 18048326 10.1073/pnas.0709483104 PMC2148333

[13] Chiu CJ, McArdle AH, Brown R, Scott HJ, Gurd FN (1970) Intestinal mucosal lesion in low flow states. Arch Surg 101: 478–483. 5457245 10.1001/archsurg.1970.01340280030009

[14] McWhinnie DL, Thompson JF, Taylor HM, Chapman JR, Bolton EM, et al (1986) Morphometric analysis of cellular infiltration assessed by monoclonal antibody labelling in sequential human renal allograft biopsies. Transplantation 42: 352–8. 3094207 10.1097/00007890-198610000-00004

[15] Greca FH, Gonçalves NM, Souza Filho ZA, Noronha L, Silva RF, et al (2008) The protective effect of methylene blue in lungs, small bowel and kidney after intestinal ischemia and reperfusion. Acta Cir Bras 23: 149–56. 18372960 10.1590/s0102-86502008000200007

[16] Zhao HD, Zhang F, Shen G, Li YB, Li YH, et al (2010) Sulforaphane protects liver injury induced by intestinal ischemia reperfusion through Nrf2-ARE pathway. World J Gastroenterol 16: 3002–10. 20572303 10.3748/wjg.v16.i24.3002 PMC2890940

[17] Wang J, Qiao L, Li Y, Yang G (2008) Ginsenoside Rb1 attenuates intestinal ischemia reperfusioninduced liver injury by inhibiting NF-κB activation. Exp Mol Med 40: 686–98. 19116454 10.3858/emm.2008.40.6.686 PMC2679344

[18] Fedorova LV, Sodhi K, Gatto-Weis C, Puri N, Hinds TD Jr, et al (2013) Peroxisome proliferator-activated receptor δ agonist, HPP593, prevents renal necrosis under chronic ischemia. PLoS One 8: e64436. 23691217 10.1371/journal.pone.0064436 PMC3654981

[19] Shah ZA, Li RC, Thimmulappa RK, Kensler TW, Yamamoto M, et al (2007) Role of reactive oxygen species in modulation of Nrf2 following ischemic reperfusion injury. Neuroscience 147: 53–9. 17507167 10.1016/j.neuroscience.2007.02.066 PMC1961622

[20] Tanaka Y, Maher JM, Chen C, Klaassen CD (2007) Hepatic ischemia-reperfusion induces renal heme oxygenase-1 via NF-E2-related factor 2 in rats and mice. Mol Pharmacol 71: 817–25. 17151289 10.1124/mol.106.029033

[21] Leonard MO, Kieran NE, Howell K, Burne MJ, Varadarajan R, et al (2006) Reoxygenation-specific activation of the antioxidant transcription factor Nrf2 mediates cytoprotective gene expression in ischemia-reperfusion injury. FASEB J 20: 2624–6. 17142801 10.1096/fj.06-5097fje

[22] Haase M, Bellomo R, Devarajan P, Schlattmann P, Haase-Fielitz A (2009) NGAL Meta-analysis Investigator Group. Accuracy of neutrophil gelatinase-associated lipocalin (NGAL) in diagnosis and prognosis in acute kidney injury: a systematic review and meta-analysis. Am J Kidney Dis 54: 1012–24. 19850388 10.1053/j.ajkd.2009.07.020

[23] Mori K, Nakao K (2007) Neutrophil gelatinase-associated lipocalin as the real-time indicator of active kidney damage. Kidney Int 71: 967–70. 17342180 10.1038/sj.ki.5002165

[24] Hjortrup PB, Haase N, Wetterslev M, Perner A (2013) Clinical review: Predictive value of neutrophil gelatinase-associated lipocalin for acute kidney injury in intensive care patients. Crit Care 17: 211. 23680259 10.1186/cc11855 PMC3672520

[25] Wang J, Qiao L, Li Y, Yang G (2008) Ginsenoside Rb1 attenuates intestinal ischemia-reperfusion- induced liver injury by inhibiting NF-kappaB activation. Exp Mol Med 40: 686–98. 19116454 10.3858/emm.2008.40.6.686 PMC2679344

[26] Guo Y, Yang T, Lu J, Li S, Wan L, et al (2011) Rb1 postconditioning attenuates liver warm ischemia-reperfusion injury through ROS-NO-HIF pathway. Life Sci 88: 598–605. 21300075 10.1016/j.lfs.2011.01.022

[27] Wu Y, Xia ZY, Dou J, Zhang L, Xu JJ, et al (2011) Protective effect of ginsenoside Rb1 against myocardial ischemia/reperfusion injury in streptozotocin-induced diabetic rats. Mol Biol Rep 38: 4327–35. 21113666 10.1007/s11033-010-0558-4

[28] Xia R, Zhao B, Wu Y, Hou JB, Zhang L, et al (2011) Ginsenoside Rb1 preconditioning enhances eNOS expression and attenuates myocardial ischemia/reperfusion injury in diabetic rats. J Biomed Biotechnol 2011: 767930. 22013385 10.1155/2011/767930 PMC3196378

[29] Hwang YP, Jeong HG (2010) Ginsenoside Rb1 protects against 6-hydroxydopamine-induced oxidative stress by increasing heme oxygenase-1 expression through an estrogen receptor-related PI3K/Akt/Nrf2-dependent pathway in human dopaminergic cells. Toxicol Appl Pharmacol 242: 18–28. 19781563 10.1016/j.taap.2009.09.009

[30] Deng C, Sun Z, Tong G, Yi W, Ma L, et al (2013) α-Lipoic acid reduces infarct size and preserves cardiac function in rat myocardial ischemia/reperfusion injury through activation of PI3K/Akt/Nrf2 pathway. PLoS One 8: e58371. 23505496 10.1371/journal.pone.0058371 PMC3591314

[31] Mao X, Wang T, Liu Y, Irwin MG, Ou JS, et al (2013) N-acetylcysteine and allopurinol confer synergy in attenuating myocardial ischemia injury via restoring HIF-1α/HO-1 signaling in diabetic rats. PLoS One 8: e68949. 23874823 10.1371/journal.pone.0068949 PMC3715528

[32] Shah ZA, Li RC, Thimmulappa RK, Kensler TW, Yamamoto M, et al (2007) Role of reactive oxygen species in modulation of Nrf2 following ischemic reperfusion injury. Neuroscience 147: 53–9. 17507167 10.1016/j.neuroscience.2007.02.066 PMC1961622

[33] Kaspar JW, Niture SK, Jaiswal AK (2009) Nrf2:INrf2 (Keap1) signaling in oxidative stress. Free Radic Biol Med 47: 1304–9. 19666107 10.1016/j.freeradbiomed.2009.07.035 PMC2763938

[34] Niture SK, Jaiswal AK (2011) INrf2 (Keap1) targets Bcl-2 degradation and controls cellular apoptosis. Cell Death Differ 18: 439–51. 20865015 10.1038/cdd.2010.114 PMC3010499

[35] Hseu YC, Chou CW, Senthil Kumar KJ, Fu KT, Wang HM, et al (2012) Ellagic acid protects human keratinocyte (HaCaT) cells against UVA-induced oxidative stress and apoptosis through the upregulation of the HO-1 and Nrf-2 antioxidant genes. Food Chem Toxicol 50: 1245–55. 22386815 10.1016/j.fct.2012.02.020

[36] Srisook K, Kim C, Cha YN (2005) Molecular mechanisms involved in enhancing HO-1 expression: de-repression by heme and activation by Nrf2, the "one-two" punch. Antioxid Redox Signal 7: 1674–87. 16356129 10.1089/ars.2005.7.1674

[37] Surh YJ, Kundu JK, Li MH, Na HK, Cha YN (2009) Role of Nrf2-mediated heme oxygenase-1 upregulation in adaptive survival response to nitrosative stress. Arch Pharm Res 32: 1163–76. 19727608 10.1007/s12272-009-1807-8

[38] Kim YM, Pae HO, Park JE, Lee YC, Woo JM, et al (2011) Heme oxygenase in the regulation of vascular biology: from molecular mechanisms to therapeutic opportunities. Antioxid Redox Signal 14: 137–67. 20624029 10.1089/ars.2010.3153 PMC2988629

